# Amelioration and deterioration: Social network typologies and mental health among female domestic workers in China

**DOI:** 10.3389/fpubh.2022.899322

**Published:** 2022-09-08

**Authors:** Binbin Tang, Mahefuzha Mamubieke, Maitixirepu Jilili, Linping Liu, Bowen Yang

**Affiliations:** School of Social and Behavioral Sciences, Nanjing University, Nanjing, China

**Keywords:** mental health, family network, friend network, face-to-face, domestic workers, coarsened exact matching, network types

## Abstract

Previous quantitative studies on the effects of social network types on mental health have obtained inconsistent or conflicting results, due to problems such as sample selection bias or crude measurement of variables. In this study, we avoided these problems by using appropriate statistical methodology to examine the effect of various forms of social network on the mental health of a sample of 987 Chinese female domestic workers. Thus, we measured social network types in terms of both network attributes (friend networks and family networks) and interaction channels (face-to-face, telephone, and WeChat/QQ channels, where the latter are two popular online messaging platforms in China), and used the coarsened exact matching method to obtain a balanced sample. The results showed that social network typologies had positive and negative effects on the mental health of this sample of domestic workers, as evidenced by (1) In terms of network attributes, family networks were associated with improved mental health and friend networks were associated with worsened mental health; (2) In terms of interaction channels, the significant amelioration in mental health from family networks came from face-to-face interactions, the significant deterioration in mental health from friends networks came from telephone interactions, and in terms of other interaction channels, family networks and friends networks had no significant effect on mental health. Robustness tests indicated that these conclusions are reliable. We discuss the possible mechanisms of which different types of social networks influence mental health.

## Introduction

Domestic workers are a large and vulnerable group in the global informal labor market, but their mental health has not received due attention (1, 2). Some studies have posited that mental health problems are prevalent among domestic workers (3), and that their psychological status is much worse than that of the general adult population (4), with this primarily manifested as high levels of anxiety and depression (5). Poor psychological health has serious consequences for domestic workers, as it can lead to suicidal thoughts or render them unable to perform their duties (5, 6). Moreover, in recent years in China, there have been several cases of elderly people or children being maltreated by mentally ill domestic workers^1^. These cases have shocked Chinese society, and serve as a warning of the urgent need to support domestic workers' mental health.

Many studies have examined the types of social networks of domestic workers and their mental health implications (4, 7–14). These studies provide us with rich insights and reveal the complexity of the impact of social networks on the mental health of domestic workers. However, this existing body of research has various limitations. First, although these studies classified social networks in terms of netwok attributes into two types of family networks and friend networks, they have neglected to examine the effects of social network in terms of interaction channels. This has generated a research gap, as the rapid development of communication technology means that people now communicate *via* an increasingly diverse array of online interaction channels, such as Facebook and WeChat/QQ (two popular online messaging platforms in China), rather than only *via* offline interaction channels (e.g., face-to-face communication); the impact of different social network types on mental health *via* these interaction channels may vary. Second, most studies have used qualitative research methods, whose conclusions are only suggestive, and the few studies that have used quantitative research methods have been based on small-scale convenience sampling methods (e.g., snowball sampling) These sampling methods may have generated selection bias, which means that the studies' conclusions may be incorrect. Thus, appropriate statistical methods must be used to solve such sampling problems and obtain valid conclusions.

To address the above-mentioned limitations of previous studies, we used a respondent-driven sampling method to collect representative sample data, a coarsened exact matching method to obtain a balanced sample, and measured social networks in terms of both its network attributes and interaction channels. This enabled us to systematically examine the relationship between various types of social network and the mental health of a sample of Chinese domestic workers.

## Literature review

Research indicates that social relations have a powerful impact on mental health (15–17), but most of these studies has examined the effects of isolated aspects of social relations, such as total network size (18, 19). Fiori et al. (20) argue that it may be more informative to examine types of social networks and their mental health implications, and according to Adams and Blieszner (21), there is likely to be considerable variation in patterns of social relations and their adaptiveness for human groups, and it should be preferable to consider this adaptiveness in terms of network typologies.

### Different attributes of social network and mental health

Research on how social networks aids the mental health of domestic workers has identified several mechanisms linking family networks and/or friend networks to these workers' mental health. First, although they had limited survey data, some studies have found that family networks (22) or friend networks (7) are important for maintaining and improving their mental health. Second, the family and friend networks has a positive impact on the mental health of domestic workers, but that the extent and conditions under which these effects occur vary. For example, Holroyd et al. (8) studied the mental health and family networks of 290 Filipino domestic workers in Hong Kong collected *via* a snowball sampling method, and found that these workers' level of contact with their relatives was inversely proportional to their probability of suffering from depression. As would be expected, in the absence of family members, social networks of friends is crucial to support mental health. Ye and Feinian (14) investigated the effects of family networks and friend networks on the self-rated health (including mental health) of 1,017 Filipino domestic workers in Hong Kong, whom they recruited *via* a multi-stage cluster sampling method. Their results showed that family networks invariably and significantly improved the mental health of these domestic workers, whereas Hong Kong-based friend networks only sometimes improved the mental health of these domestic workers, while non-Hong Kong-based friend networks had no effect. Moreover, there was an interdependence between the effects of a family networks and a friend networks. Similarly, Iyer et al. (3) interviewed 30 foreign domestic workers in Singapore, and van der Ham et al. (23) studied 500 domestic workers in the Philippines who were recruited *via* snowball sampling, and both found that family contact was particularly beneficial for the mental health of domestic workers, whereas friend contact was either beneficial or had no effect. In contrast, in an empirical study of 261 female Filipino domestic workers in Macau, which was conducted using a snowball sampling method, Mendoza et al. (10) found that from both family and friend networks did not have a significantly positive impact on domestic workers' mental health, and in some cases worsened their mental health.

The reason for the inconsistent and contradictory results described above has not been determined, but a possible explanation is that these studies have largely failed to distinguish how the impacts of social network types on domestic workers' mental health vary with the channels of interaction. For example, Ye and Feinian (14) used a measure of family and friends networks that consisted of three interaction channels: face-to-face, telephone, and Internet. However, despite the fact that the impact of social network types on mental health may vary between different interaction channels, the authors (in common with other studies) combined social network types from all three interaction channels into a homogeneous result, which may have led to erroneous interpretations. Therefore, it is necessary to explore the relationship between social network types and mental health in each interaction channel separately.

### Different interaction channels of social network and mental health

The development of communication technology means that people now interact in range of ways, such as *via* face-to-face interaction, which is constrained by geographical location, and telephone and Internet social network interaction, which are unconstrained by geographical location. Most studies have suggested that face-to-face communication is the primary method of interpersonal interaction, and that this is supplemented by telephone and Internet-based social networking (24, 25). However, several studies have posited that telephone communication is more important than face-to-face communication, in that as a person's interpersonal relationships mature and increase, these are maintained by distance-independent telephone interaction rather than by distance-dependent face-to-face interaction (26, 27). Overall, it is clear that offline and online communication modes—from face-to-face to telephone and Internet social media modes—encompass diverse channels of interaction that maintain, strengthen, and develop social networks.

Studies examining the relationship between social network types *via* various interaction channels and mental health have suggested that only face-to-face network can diminish depression (25) and loneliness (28, 29), because of the uniquely pleasurable and relaxing nature of face-to-face communication. However, it was also argued that the instantaneity of telephone communication provides rapid social connection and thus emotional benefits (26). Other studies have found that the impact on mental health of social network established *via* Internet interactions is complex, and may be negative (30, 31) or positive (32, 33).

However, the above studies were based on surveys of college students, and thus may not be generalizable to domestic workers. Specifically, unlike college students, domestic workers have a mobile and isolated working environment, in which they may have few face-to-face interactions with family and friends. Thus, domestic workers may find telephone and social media to be more convenient ways to interact with family and friends. Moreover, two studies on female domestic workers in Singapore have shown that compared with not using mobile phones and Internet social media to interact with family and friends, using such methods was associated with these workers having more social contact and significantly improved mental health (9, 34). However, these studies have not compared the effects on mental health of social network types received through different interaction channels.

It is clear that there is a dearth of quality research on the effects of social network types on the mental health of domestic workers. First, research on the relationship between social network typologies and mental health has used imprecise methods, and few studies have compared the effects of social network types *via* different interaction channels on mental health. Thus, more research is needed to determine the validity of previous studies' findings. Second, domestic workers do not randomly decide whether to interact offline (i.e., face-to-face) and/or online (*via*, for example, Facebook or WeChat/QQ) with family and friends; their decision is guided by constraints. These include the barrier of geographical distance and the “digital divide” of communication technology, and imply that the frequency of interactions *via* offline and/or online modes is biased by domestic workers' circumstances or technological skills, which leads to non-randomness in the way and frequency of interactions across different social network types of domestic workers. Consequently, without the careful balancing of a study sample, there is a high risk of bias in sample selection and thus also in study findings. This underscores the need for the balanced matching of samples to obtain unbiased estimates, which we have addressed in this study.

## Methods

### Data

We used data from a domestic workers survey conducted in four cities in China (Nanjing, Wuxi, Guangzhou, and Foshan) in 2019, which employed respondent-driven sampling to obtain a total of 1,007 valid samples (35) The questionnaire collected information on domestic workers' basic situation, employment relationship, job attitudes, job training, and mental health. We excluded male domestic workers as the domestic service industry is dominated by women, and our final valid sample comprised 987 female domestic workers. Domestic workers as those who perform paid domestic work in private households, excluding part-time workers who occasionally perform domestic work (36).

### Measurements

#### Dependent variable

Our dependent variable is the depressive symptoms of domestic workers, measured by the 10-item Center for Epidemiological Studies Depression Scale developed by Radloff (37). This scale shows good reliability and validity in assessing the mental health of specific groups, such as the elderly and adolescents, and the general population (38, 39). In each item of the scale, respondents are asked to note how often they have experienced a negative mental state in the previous week. The options are “never or very infrequently (<1 day),” “not much (1–2 days),” “sometimes or half of the time (3–4 days),” and “most of the time (5–7 days).” We allocated a response of “never or very infrequently (<1 day)” a score of 0, a response of “not much (1–2 days)” a score of 1, a response of “sometimes or half of the time (3–4 days)” a score of 2, and a response of “most of the time (5–7 days)” a score of 3. As the positive emotion items “hopeful about the future” and “feeling very happy” are reverse questions, we also reversed the values. The reliability of the scale is 0.782 (Cronbach's α), indicating higher internal consistency. We used factor analysis to extract a common factor (Kaiser–Meyer–Olkin = 0.839) and standardized the results to obtain a depressive tendency score from 0 to 100, where a higher score indicates worse mental health.

#### Independent variables

The core independent variable is the respondent's social network. Domestic workers communicate and interact with family members and friends through various channels to obtain encouragement, comfort, love, empathy, and care (40), which are especially important for domestic workers in bad working conditions. We used a questionnaire that asks respondents how often they interacted with their family and friends in 2018 *via* face-to-face meetings, phone calls, and WeChat/QQ. The options are “every day,” “several times a week,” “once a week,” “at least once a month,” “at least once every 6 months,” and “once a year.” We first classified social networks into family networks and friends networks by network attributes, followed by further subdivide family networks and friends networks into face-to-face interaction networks, telephone interaction networks, and WeChat/QQ interaction networks by different interaction channels. Where the interaction strength of different network types is measured by contact frequency.

Accordingly, we allocated a response of “every day” a score of 6, a response of “several times a week” a score of 5, a response of “once a week” a score of 4, a response of “at least once a month” a score of 3, a response of “at least once every 6 months” a score of 2, a response of “once a year” a score of 1, and a response of “no contact at all” a score of 0, so a higher score indicated that the respindent interacts more frequently with family members or friends. As the questionnaire asks about the frequency of interaction between a respondent and multiple family members (e.g., the interaction between a respondent and her parents, spouse, or children), this was regarded as an indicator of the comprehensive interaction frequency between a respondent and their family members, according to the following equation:


(1) $$\begin{array}{l} E_{i}=\left(\sum S_{ij}\right)/N \end{array}$$

Where *E*_*i*_ is the comprehensive interaction frequency between respondent *i* and her family *j* (her parents, spouse, first child, second child, and youngest child); *S*_*ij*_ is the frequency of interaction between respondent *i* and family *j*, scored from 0 to 6; and *N* is the number of people in her family. This afforded a comprehensive frequency of interaction between respondents and their families, which ranged from 0 to 6.

We also controlled for three categories of variables that affect mental health: demographic variables, employment-related variables, and other variables. The demographic variables are age, education level, marriage, and hukou (household registration); the employment-related variables are the nature of the job, work years, weekly rest days, monthly income, employer-installed monitoring equipment, job injury, and chronic disease; and the other variables are religious belief and whether a respondent seeks help when experiencing problems. These variables are also described below in Table 1.

**Table 1:** Description of model variables (*N* = 987).

**Variables**	**Category/indicator**	**Percentage/value**	**Min**	**Max**
Dependent variable				
Depressive tendency score	Mean (standard deviation)	16.60 (16.74)	0	100
Core independent variable				
Social network of different attributes				
Frequency of contact with family members	Mean (standard deviation)	4.52 (1.03)	0	6
Frequency of contact with friends	Mean (standard deviation)	4.29 (1.61)	0	6
Social network through different channels				
Face-to-face with family	Mean (standard deviation)	3.98 (1.47)	0	6
Call your family	Mean (standard deviation)	4.66 (1.35)	0	6
Contact your family on WeChat /QQ	Mean(standard deviation)	4.78 (1.89)	0	6
Face-to-face with friends	Mean (standard deviation)	4.16 (2.07)	0	6
Call your friends	Mean (standard deviation)	3.63 (1.91)	0	6
Contact your friends on WeChat /QQ	Mean (standard deviation)	4.48 (2.07)	0	6
Control variables				
Work years	Mean (standard deviation)	8.20 (6.82)	0.10	38.60
Age	Under the age of 50	42.45%	0	1
	Age 50 and above	57.55%		
Education Level	Primary and below	35.16%	1	3
	Junior high school	49.65%		
	High school	15.20%		
Marriage	In the wedding	88.75%	0	1
	Not in marriage	11.25%		
Hukou	Rural registered permanent residence	73.86%	0	1
	Urban hukou	26.14%		
Monthly income	3,000 yuan or less	19.25%	0	1
	More than 3,000 yuan	80.75%		
Nature of work	Care work	60.89%	0	1
	Non-care work	39.11%		
Weekly rest days	Don't rest	28.57%	1	3
	Only 1 day off	59.98%		
	2 days off	11.45%		
Employer installs camera	The installation	25.84%	1	3
	Not to install	63.63%		
	Don't know	10.54%		
Job injury	Yes	11.04%	0	1
	No	88.96%		
Ask for help in the past 12 months	Yes	25.53%	0	1
	No	74.47%		
Religious beliefs	Yes	12.77%	0	1
	No	87.23%		
Chronic diseases	Yes	47.11%	0	1
	No	52.89%		

### Research methods

#### Coarsened exact matching

Domestic workers choose to interact with family and friends face-to-face or phone calls and Wechat/QQ, which are not random choices. The “digital divide” in communication technology mean that the frequency of different interaction modes is biased, which leads to the self-selection in the frequency of interactions across different social network types. If we do not deal with the problem of sample balance, there is a high probability that there will be sample selectivity deviation, the conclusion may be biased. So, It is necessary to balance the samples to obtain unbiased estimation. Sample matching is one of the important methods to reduce selectivity bias. Based on the idea of covariate matching, Iacus et al. (41) proposed the CEM method, which attempts to solve the self-selection problem by selecting similar samples from survey data to simulate the effect of randomization experiments. Compared with traditional matching methods, such as propensity score and Mahalanobis matching, the advantages of the CEM method include: (1) it does not require high prior sample size requirements for the observed data; (2) the matching process is simpler, with the researcher selecting the matching level based on specific, intuitive and substantive information; (3) the equilibrium constraint of a covariate can be studied and improved individually. Therefore, CEM is considered a line of defense to protect causal inferences from threats to validity.

According to the data, 6 percent of domestic workers do not have smartphones and 10 percent do not know how to use Wechat /QQ, popular social software, which provides more convenience and possibility for the interpersonal interaction between respondents and their family and friends. However, the ability to own a smartphone and use of smartphone applications such as WeChat/QQ is a non-random event which is affected by a series of social and economic factors such as age, income and educational level etc.

Moreover, our other data obtained from our sample revealed that the respondents' average age was 50 years (with the oldest being 71 years); the average monthly income is RMB 4,905, with a minimum monthly wage of RMB 300 and a maximum monthly wage of RMB 17,800; nearly 20% had an average monthly income of <3,000 RMB, more than 73% had a rural hukou, and 80% had a junior high school education or below. These data show that compared with employees in other sectors, these domestic workers were older, and had lower living standards and education levels; such a subset of society has significantly less ability to use new communication technologies than others in society. Therefore, we applied the CEM method developed by Iacus et al. (41, 42) to match the covariables affecting the use of WeChat/QQ, and used the resulting matched and balanced data for regression analyses, to eliminate the deviations caused by the raw data.

The basic operation of the CEM method consists of three steps: the first is to coarse-code variables; the second is to apply an exact matching algorithm to the coarse data to generate weighted variables and matching units; and the third is to retain the successfully matched data, discard the unmatched data, and make use of the weights for subsequent regression analysis. Thus, the ideality of the data matching can be determined by the multivariate imbalance index indicator (L_1_) (42). The value of L_1_ is calculated based on the data and the selected covariables, and ranges from 0 (completely balanced) to 1 (completely unbalanced); thus, a larger value indicates a greater imbalance between the groups under comparison. Well-matched data result in a significant reduction in L_1_, which is calculated as follows:


(2) $$\begin{array}{l} L_{1\left(f.g\right)}=\frac{1}{2}\sum \varepsilon_{1}......\varepsilon_{k}|f_{\varepsilon_{1}......\varepsilon_{k}}-g_{\varepsilon_{1}......\varepsilon_{k}}| \end{array}$$

Equation (2) indicates that the covariables after coarsening are first cross-tabulated as X_1_ × ...... × X_k_ for the treated and the control groups, respectively. Then, the *k-*dimensional relative frequency for the treated group is recorded in *f*_ε1......ε*k*_ units and that for the control groups is recorded in *g*_ε1......ε*k*_ units. The absolute difference between all unit values is the L_1_ value (43). If the two groups of data are completely separated, then L_1_ = 1, whereas if the two groups of data are completely overlapping, then L_1_ Equals = 0.

## Results

### Matching result analysis

Table 2 reports the balance distribution of covariables affecting WeChat/QQ usage before and after matching. Model 1 shows that before matching, age, education level, and marital status significantly affected the probability of WeChat/QQ usage. That is, compared with domestic workers who were aged 50 or older, did not have a junior high school education or above, and were not married, domestic workers who were aged younger than 50, had a junior high school education or above, and were married were more likely to know how to operate social media software such as WeChat/QQ. Model 2 shows that the CEM method largely preserved the data sample size (i.e., removed only 103 samples) and improved the balance distribution of covariables affecting WeChat/QQ usage (*p* > 0.05). Thus, matching afforded a well-balanced random sample.

**Table 2:** Covariable balance results of WeChat /QQ usage before and after matching.

	**Before matching (Model 1)**	**After matching (Model 2)**
Age (0 = under 50)	−2.177^***^ (0.403)	0.0063 (0.237)
Education level (0 = primary school and below)		
Junior high school	1.239^***^ (0.260)	−0.053 (0.239)
High school and above	2.416^***^ (0.631)	−0.091 (0.462)
Marriage (0 = not married)	0.661^*^ (0.307)	0.031 (0.478)
Monthly income (0 = 3,000 yuan and below)	0.315 (0.262)	0.335 (0.259)
Household Registration (0 = Urban)	−0.155 (0.304)	0.209 (0.293)
Constant term	2.115^**^ (0.666)	1.469^*^ (0.705)
Sample size	987	884
R2	0.19	0.01

Robust standard error in parentheses.

* P < 0.05,

** P < 0.01,

*** P < 0.001.

Table 3 reports the results for L_1_ based on CEM. Before matching, age, education level, and marital status were the variables that significantly influenced WeChat/QQ use, with L_1_ values of 0.4, 0.4, and 0.1, respectively, and thus the comprehensive multivariate imbalance index L_1_ was almost equal to 0.45. After matching, the L_1_ values of a single-variable index and those of a multivariate index after synthesis were almost equal to 0. This indicates that the data were significantly less imbalanced after matching, showing that excellent matching was achieved.

**Table 3:** Imbalance index L_1_ before and after CEM.

	**Before matching: L_1_ (average)**	**After matching: L_1_ (average)**
Age (0 = under 50)	0.398 (−0.389)	1.0 e-15 e-15 (1.3e-15)
Fixed number of year of the education	0.361 (0.494)	1.1 e-15 (4.4 e-16)
In marriage (0= not in marriage)	0.095 (0.095)	1.1 e-16 (−1.1 e-16)
Multivariate L_1_	0.441	9.281 e-16
Sample size	987	884

The mean difference of L_1_ in parentheses.

### Regression analysis

We next used the matched samples for regression analysis. As the dependent variable—the depressive symptoms of domestic workers, as a marker of their mental health—is a continuous variable, a multiple linear regression model was applied to investigate the effects of social network types from different attributes and *via* different interaction channels on the mental health of domestic workers. Table 4 below reports the regression results for a set of nested regression models.

**Table 4:** Regression results of social network with different network attributes on mental health of domestic workers.

	**Model 3**	**Model 4**	**Model 5**
Age (0 = under 50)	−0.673 (1.197)	−0.895 (1.196)	−1.198 (1.200)
Education level (ref = Primary school and below)			
Junior high school	−2.734^*^ (1.191)	−2.633^*^ (1.188)	−2.652^*^ (1.185)
High school and above	−3.041 (2.278)	−2.628 (2.276)	−2.399 (2.272)
Married	−1.927 (2.338)	−1.137 (2.349)	−1.270 (2.344)
Monthly income (0 = 3,000 yuan and below)	−3.260^*^ (1.412)	−3.490^*^ (1.410)	−3.258^*^ (1.409)
Hukou (ref = Urban)	0.00711 (1.407)	0.367 (1.409)	0.591 (1.409)
Religious belief (0 = none)	0.813 (1.688)	1.358 (1.695)	1.354 (1.691)
Chronic diseases (0 = none)	6.449^***^ (1.116)	6.602^***^ (1.113)	6.698^***^ (1.111)
Job injury (0 = none)	7.113^***^ (1.763)	6.522^***^ (1.772)	6.490^***^ (1.767)
Ask for help when encountering a problem (0 = none)	4.428^***^ (1.284)	4.207^**^ (1.282)	4.122^**^ (1.279)
Working fixed number of year	−0.0288 (0.0789)	−0.0190 (0.0787)	−0.0286 (0.0786)
Caregiving work (0 = non-caregiving)	0.868 (1.167)	0.540 (1.170)	0.768 (1.171)
Weekly rest days (0 = no rest)			
Only 1 day off	−0.847 (1.205)	−0.478 (1.209)	−0.550 (1.206)
2 days off	−0.557 (1.914)	0.171 (1.927)	−0.0794 (1.925)
Employer installs camera (0 = Install)			
Not to install	−2.027 (1.241)	−2.257 (1.240)	−2.410 (1.238)
Don't know	−0.114 (1.994)	−0.0837 (1.988)	0.0462 (1.983)
Frequency of contact with family members		−1.479^**^ (0.563)	−1.802^**^ (0.578)
Frequency of contact with friends			0.824^*^ (0.347)
Constant term	19.76^***^ (3.173)	25.77^***^ (3.903)	23.84^***^ (3.976)
Sample size	884	884	884
R2	0.112	0.119	0.125

Robust standard error in parentheses.

* P < 0.05,

** P < 0.01,

*** P < 0.001.

Model 3 in Table 4 is the basic model, which reports the influence of a series of control variables on the mental health of domestic workers. The model shows that the mental health of domestic workers with a higher education level and higher monthly income was significantly better than that of domestic workers with a lower education level and lower monthly income, under the assumption that other control variables were unchanged. Domestic workers who suffered from chronic diseases or had been injured physically or psychologically at work (due to, for example, assault, abuse, harassment, or complaints) had significantly worse mental health than those who did not suffer from chronic diseases or had not been injured physically or psychologically at work. These findings are consistent with common expectations, and the fact that the mental health of domestic workers who had sought help was worse than that of those who had not.

Models 4 and 5 include social network variables. Model 4 includes family network variables, based on Model 3, and the regression results for Model 4 show that contact with family had a significantly positive effect on the mental health of domestic workers, at significance level of 0.05. This means that the more frequently domestic workers are in contact with their families, the better was their mental health. Model 5 includes friend network variables, based on Model 4. The results for Model 5 clearly demonstrates that frequent contact with friends significantly worsened the mental health of domestic workers, and that after controlling for family contact. These findings are consistent with the mainstream view in previous studies: that family networks invariably improves a person's mental health, and the relationship between the two variables is relatively stable, whereas friend networks may worsen a person's mental health (3, 14, 23).

As the effects of social network on mental health vary with the attributes of a network, the regression model in Table 5 examines the interactive channels *via* which social network affects domestic workers' mental health.

**Table 5:** Regression results of social network in different interactive channels on mental health of domestic workers.

	**Model 6**	**Model 7**	**Model 8**	**Model 9**
Age (0 = under 50)	−0.953 (1.204)	−0.977 (1.197)	−0.856 (1.200)	−1.182 (1.208)
Education level (1 = Primary school and below)				
Junior high school	−2.807^*^ (1.190)	−2.687^*^ (1.189)	−2.750^*^ (1.188)	−2.803^*^ (1.189)
High school and above	−2.933 (2.275)	−2.683 (2.274)	−2.846 (2.275)	−2.596 (2.273)
Marriage (0 = not married)	−1.505 (2.355)	−1.742 (2.335)	−1.799 (2.333)	−1.096 (2.352)
Monthly income (0 = 3,000 yuan and below)	−3.339^*^ (1.419)	−3.209^*^ (1.407)	−3.103^*^ (1.410)	−3.358^*^ (1.417)
Hukou (0 = Urban)	0.761 (1.446)	0.0970 (1.403)	−0.0949 (1.404)	0.676 (1.446)
Religious belief (0 = none)	1.054 (1.692)	0.813 (1.685)	1.002 (1.697)	1.258 (1.696)
Chronic diseases (0 = none)	6.574^***^ (1.116)	6.743^***^ (1.116)	6.640^***^ (1.116)	6.988^***^ (1.120)
Job injury (0 = none)	6.739^***^ (1.775)	7.081^***^ (1.758)	6.816^***^ (1.763)	6.447^***^ (1.773)
Ask for help when encountering a problem (0 = none)	4.286^***^ (1.283)	4.307^***^ (1.283)	4.477^***^ (1.281)	4.237^***^ (1.283)
Working fixed number of year	−0.0248 (0.0788)	−0.0466 (0.0788)	−0.0341 (0.0791)	−0.0391 (0.0790)
Caregiving work (0 = non-caregiving)	0.598 (1.182)	1.101 (1.170)	1.023 (1.166)	0.824 (1.187)
Weekly rest days (0 = no rest)				
Only 1 day off	−0.419 (1.226)	−1.085 (1.204)	−0.824 (1.216)	−0.522 (1.232)
2 days off	0.553 (2.014)	−1.042 (1.914)	−0.600 (1.918)	0.304 (2.011)
Employer installs camera (0 = Install)				
Not to install	−2.267 (1.244)	−2.184 (1.239)	−2.261 (1.246)	−2.563^*^ (1.249)
Don't know	−0.174 (1.993)	−0.148 (1.987)	−0.0722 (1.990)	−0.285 (1.988)
Frequency of face-to-face with family	−0.839^*^ (0.412)			−0.814^*^ (0.421)
Frequency of face-to-face with friends	0.292 (0.263)			−0.079 (0.300)
Frequency of call your family		−0.713 (0.417)		−0.559 (0.425)
Frequency of call your friends		0.872^**^ (0.303)		0.805^*^ (0.344)
Frequency of contact your family on WeChat /QQ			−0.790^*^ (0.344)	−0.546 (0.359)
Frequency of contact your friends on WeChat /QQ			0.641^*^ (0.305)	0.394 (0.348)
Intercept	21.43^***^ (3.567)	20.04^***^ (3.637)	20.54^***^ (3.389)	23.29^***^ (3.944)
Sample size	884	884	884	884
R2	0.118	0.121	0.119	0.128

Robust standard error in parentheses.

* P < 0.05,

** P < 0.01,

*** P < 0.001.

Model 6 reports the effect of face-to-face social network types on the mental health of domestic workers. The regression results show that when control variables were included, face-to-face contact with family members significantly improved the mental health of domestic workers (at a significance level of 0.05). That is, the more that domestic workers interacted face-to-face with family members, the better was the domestic workers' mental health. In contrast, face-to-face interaction with friends had a negative effect on domestic workers' mental health, but this result was not statistically significant (*p* > 0.05). Model 7 reports the effect of the type of social network through telephone interactions on the mental health of domestic workers. The regression results show that telephone interaction with family members somewhat improved domestic workers' mental health, but this result was not statistically significant (*p* > 0.05). In contrast, telephone interaction with friends consistently significantly worsened domestic workers' mental health (*p* < 0.01), with the frequency of domestic workers' telephone interactions with friends being inversely proportional to these workers' mental health. Model 8 examines the effect of the type of social network through WeChat/QQ interactions with family members and friends on the mental health of domestic workers. The regression results show that domestic workers' mental health was improved by WeChat/QQ interactions with family members but worsened by WeChat/QQ interactions with friends. Both of these effects were significant (*p* < 0.05).

However, domestic workers may also interact with family networks and friend networks through three channels simultaneously: face-to-face, by telephone, and by WeChat/QQ. This is explored by a comparison of the results from Model 9 with those for Models 6, 7, and 8, which indicates that face-to-face interactions with family members consistently and significantly improved the mental health of domestic workers (*p* < 0.05); face-to-face interactions with friends also improved the mental health of domestic workers, but this effect was not significant (*p* > 0.05). In addition, there was no change in the direction of the effect of telephone interactions with family networks or friend networks on domestic workers' mental health, and telephone interactions with friends invariably significantly worsened domestic workers' mental health (*p* < 0.05). In addition, family networks or friend networks *via* WeChat/QQ interactions no longer had a significant effect on the mental health of domestic workers (*p* > 0.05).

### Robustness analysis

We adopted two methods for testing the robustness of the identified relationships between domestic workers' social network typologies and their mental health. The first method requires the construction of a new dependent variable. Radloff (37) stated that the 95^th^ percentile corresponding to the total score of the 10-item Center for Epidemiological Studies Depression Scale can be used as the threshold of depression. Accordingly, we constructed a dependent variable of depressive symptoms, which when scoring <13 (indicating the absence of depressive symptoms) is allocated a value of 0 and when scoring ≥13 (indicating the presence of depressive symptoms) is allocated a value of 1. The second method is to perform testing with the original (i.e., non-matched and thus unbalanced) sample. These test results are shown in Table 6, and indicate that irrespective of whether the test model uses a new dependent variable (the first method) or a new sample (the second method) for regression analysis, the test results of Model 1 and Model 3 are consistent with those for Model 5 in Table 4, and the test results of Model 2 and Model 4 are also substantially consistent with those for Model 9 in Table 5. These robustness tests therefore confirm the reliability of the results of our regression analysis.

**Table 6:** Robustness test results.

**Test method 1 (Dependent variable: depressive tendency)**		**Test method 2 (Dependent variable: mental health)**	
**Test model 1 (*****N*** **=** **884)**	**Regression coefficient**	**Test model 3 (*****N*** **=** **987)**	**Regression coefficient**
Frequency of contact with family members	−0.014^+^ (0.008)	Frequency of contact with family members	−1.542** (0.543)
Frequency of contact with friends	0.013** (0.005)	Frequency of contact with friends	0.738* (0.331)
**Test model 2 (*****N*** **=** **884)**		**Test model 4 (*****N*** **=** **987)**	
Frequency of face-to-face with family	−0.012* (0.006)	Frequency of face-to-face with family	−0.936* (0.399)
Frequency of face-to-face with friends	0.004 (0.004)	Frequency of face-to-face with friends	−0.037 (0.285)
Frequency of call your family	0.002 (0.005)	Frequency of call your family	−0.185 (0.417)
Frequency of call your friends	0.011* (0.005)	Frequency of call your friends	0.621^+^ (0.327)
Frequency of contact your family on WeChat /QQ	−0.003 (0.005)	Frequency of contact your family on WeChat /QQ	−0.563 (0.344)
Frequency of contact your friends on WeChat /QQ	0.001 (0.005)	Frequency of contact your friends on WeChat /QQ	0.331 (0.330)

The significance level: + p < 0.1, *p < 0.05, **p < 0.01, ***p < 0.001.

Standard error in brackets.

Original control variables are included.

The dependent variables of test model 1 and Model 2 were dummy variables of depression tendency, and the sample size was 884 after matching with balance problems.

The dependent variable of test model 3 and model 4 was the continuous variable of depressive tendency score, and the sample was the pre-matching sample without dealing with the balance problem, with a sample size of 987.

## Discussion

This study explored the relationship between social network typologies and the mental health of domestic workers, after effectively solving the sample balance problem using the CEM method. The results showed that different types of social network had distinct and important effects on the mental health of domestic workers in our sample. For example, the mental health of domestic workers was consistently improved by family networks but consistently worsened by friend networks. We also found that when other forms of interpersonal interaction were not controlled for, the mental health of domestic workers was significantly improved by face-to-face interaction and WeChat/QQ interaction with family members, whereas it was significantly worsened by telephone interaction and WeChat/QQ interaction with friends.

However, with control for other types of interpersonal interaction, the intermediary effect of WeChat/QQ on the mental health status of domestic workers was found to be complicated. Thus, although it was again revealed that domestic workers' mental health was improved by telephone and WeChat/QQ interaction with their family network, this effect was not significant (*p* > 0.05). Similarly, although domestic workers' mental health was again worsened by telephone and WeChat/QQ interaction with their friend network, the WeChat/QQ interaction was not significant (*p* > 0.05). Finally, we found that social network *via* the most elementary form of interaction—face-to-face interaction, with either a family network or a friend network—was highly effective in improving the mental health of domestic workers, although this result was not significant at the level of 0.05 for the friend network. The results of robustness tests confirmed the validity of these results.

It is intriguing that domestic workers' mental health was improved by family networks and yet worsened by friend networks. This may be attributable to a woman's family network being a more intimate core network than her friend network, leading to the former network having more natural and stable social ties than the latter network. Moreover, compared with a friend network, there may be no competition and conflict in a family network and thus a more reasonable allocation of resources. Furthermore, domestic workers primarily work, day after day and year after year, to earn money to support their families, and are supported by their families during employment-related periods of isolation *via* reverse remittance, emotional feedback, and the provision of a virtual family resource.

A family network is also an interdependent network of intimate relationships, and the support supplied to domestic workers by their family network is invariably selfless and helpful. In contrast, a friend network consists of unstable social tie-based networks, its availability is affected by the addition of new friends and the departure of old friends, and the relationship intimacy it supplies to domestic workers depends on the number of relevant resources it contains. As a consequence, the value of a friend network to a domestic worker varies with the network's level of relationship intimacy, and there is always a limit to the friend network-based support that domestic workers can obtain. Thus, friend networks do not necessarily improve and can even worsen the mental health of domestic workers, especially if such a network contains conflict.

In addition, it is clear that the impact on domestic workers' mental health of face-to-face, telephone, and WeChat/QQ interactions with family networks or friend networks varies with the attributes of these networks. Previous studies comparing these three channels of interaction have found that face-to-face interaction generates stronger and more effective ties within social networks than telephone or WeChat/QQ interactions, thus leading to the greatest improvements in mental health. In addition, from the perspective of media richness theory, face-to-face interaction is one of the richest communication media: it is a “real presence” that enables real-time interactive feedback, multi-threading, and spoken language performance, and thus enables more focus on individual capture than other interaction methods. Face-to-face interaction also effectively reduces the uncertainty and ambiguity in communication, and thus develops the best interactive relationships. In contrast, due to the “absence” or “unreal presence” of telephone and WeChat/QQ interactions, factual information is used to effectively reduce the uncertainty in communication, which is more suitable for task-oriented communication than emotion-oriented communication (44). Thus, compared with intermediary interaction *via* a telephone or WeChat/QQ, direct (i.e., face-to-face) interaction is the most efficient way for domestic workers to build and maintain close relationships. In sum, meeting with family and friends develops and maintains domestic workers' close relationships, and having a close social network invariably has positive effects on domestic workers, such as improving their mental health.

We also noticed that family networks and friend networks had differing effects on the mental health of domestic workers. We believe that that this can be accounted for by the concept of “network privatism” proposed by Campbell (45). This concept holds that social networks are diverse, with both strong and weak connections, and that a mediating interaction is more beneficial for strong-relationship networks than for weak-relationship networks. This alters the balance between strong and weak relationships, and thus when intimate relationships are important, individuals' social networks converge with strong networks (comprising strong ties) rather than with broad networks (comprising weak ties). Thus, this mediating interaction has positive consequences for strong networks and negative consequences for weak networks. For domestic workers, family networks are strong relational networks compared to friend networks.

## Conclusion

This study shows that it is crucial for Chinese domestic workers to maintain their mental health by fostering a good social network consisting of intimate relationships. First, our findings indicate that when constructing social networks to improve their mental health, domestic workers should focus on the development of a family ties-based network. Such a network consists of natural intimate relationships that can effectively alleviate domestic workers' mental health problems, regardless of how they interact with family members. Second, our findings show that face-to-face interactions are an effective channel *via* which domestic workers can improve their mental health, either from a family network or a friend network.

The two other key contributions of this study are as follows. First, we overcome the previously intractable or neglected problem of obtaining a balanced sample by judiciously employing statistical methods. Second, we enrich the literature by determining that social network both ameliorates and deteriorates domestic workers' mental health, as shown by exploratory analysis and a comparison of the effects of various types of social network, *via* different channels, on domestic workers' mental health. However, due to limitations, these conclusions are based on cross-sectional data, and thus must be validated in future work on larger samples. We therefore expect that future studies will enrich this research area by using more sophisticated measurements and longitudinal analyses.

## Data availability statement

The data analyzed in this study is subject to the following licenses/restrictions: The data that support the findings of this study are available from corresponding author upon reasonable request. Requests to access these datasets should be directed to linpingl@163.com.

## Author contributions

BT and MM undertook completing the main draft, including conceived and proposing research methods, data analysis, and manuscripts writing. MJ and BY completed editing manuscript and draft review. LL is the primary investigator and lead researcher on this study. All authors are accountable for the content of the work. All authors contributed to the article and approved the submitted version.

## Funding

This study was funded by the Key Project of National Social Science Fund of China (Grant-No. 18ASH007) and the Program B for Outstanding Ph.D. Candidate of Nanjing University (Project No. 201902B058).

## Conflict of interest

The authors declare that the research was conducted in the absence of any commercial or financial relationships that could be construed as a potential conflict of interest.

## Publisher's note

All claims expressed in this article are solely those of the authors and do not necessarily represent those of their affiliated organizations, or those of the publisher, the editors and the reviewers. Any product that may be evaluated in this article, or claim that may be made by its manufacturer, is not guaranteed or endorsed by the publisher.

## Footnotes

^1^Red Star News, 2020, “67-year-old nanny who suffocated 83-year-old was informal hospital nurse, and her husband said she had suffered from mental illness,” May 13, 2020. URL: https://baijiahao.baidu.com/s?id=1666580543214174211&wfr=spider&for=pc%20 (in Chinese) [Accessed June 4, 2022]. Beiqing.com, 2021, “ 'Mentally abnormal' nanny abuses the elderly, a home care company is sentenced to liability,” August 5, 2021. URL: https://t.ynet.cn/baijia/31225673.html%20 (in Chinese) [Accessed June 5, 2022]. China.com, 2017, “A nanny and a employer fight for a child, the woman once suffererd from mental illness and the housekeeping company also know”, July 5, 2017. Available at: https://news.china.com/socialgd/10000169/20170705/30917540.html%20 (in Chinese) [Accessed June 5, 2022].
